# Training improves the handling of inhaler devices and reduces the severity of symptoms in geriatric patients suffering from chronic-obstructive pulmonary disease

**DOI:** 10.1186/s12877-020-01804-4

**Published:** 2020-10-09

**Authors:** Marie-Christine Luley, Tobias Loleit, Elmar Knopf, Marija Djukic, Carl-Peter Criée, Roland Nau

**Affiliations:** 1 Department of Geriatrics, Protestant Hospital Göttingen-Weende, An der Lutter 24, 37075 Göttingen, Germany; 2 Institute of Neuropathology, University Medical Center Göttingen, Göttingen, Germany; 3 Department of Pneumology, Protestant Hospital Göttingen-Weende, Göttingen, Germany

**Keywords:** Chronic-obstructive pulmonary disease - Inhaler devices, Geriatrics, Compliance

## Abstract

**Purpose:**

Elderly patients with impaired vision, cognitive decline or motor/sensory disturbances of their fingers suffering from chronic-obstructive pulmonary disease (COPD) encounter difficulties in handling inhaler devices used as the cornerstones of treatment of pulmonary obstruction. Many elderly patients make severe mistakes which impede adequate drug delivery to the bronchioles. This multimodal training program was designed to reduce the number of handling mistakes of inhaler devices.

**Methods:**

From October 1, 2016 to September 30, 2017, a prospective intervention study was conducted in 38 in-patients > 65 years (median age 79 years) with previously diagnosed COPD. The effect of an 8-day intervention comprising daily counselling and video demonstration according to the recommendations of the German Airway League on the frequency of mistakes during handling of inhaler devices, the forced expiratory volume in 1 s (FEV1), the forced vital capacity (FVC) and the perception of symptoms (COPD Assessment Test, CAT) were studied. Measurements on days 1 and 8 were compared by Wilcoxon signed rank test.

**Results:**

The number of handling mistakes per patient decreased as a consequence of the intervention from 3.0 (0–7) to 0.5 (0–6) [median (minimum-maximum; *p* < 0.0001)]. The CAT Score decreased from 19.5 (14/24) to 14.5 (10.75/21) [median (25./75. percentile; *p* < 0.0001) indicating a substantial reduction of clinical symptoms. Conversely, FEV1 and FVC only slightly increased (difference statistically not significant). At study entry, the number of handling mistakes was inversely correlated with the Mini Mental Status Test (MMST) score (*p* = 0.01). The reduction of the number of handling mistakes during the intervention was not correlated with the MMST.

**Conclusion:**

In COPD, intensive training for 8 days improved the handling of inhalers and reduced clinical symptoms in geriatric patients. Patients with cognitive abnormalities also benefitted from this intervention.

**Trial registration:**

German Clinical Trials Registry DRKS00023196, date of registration September 29, 2020 (retrospectively registered).

## Background

Worldwide, the prevalence of stage II or more severe chronic-obstructive pulmonary disease (COPD) is 10.1% with a greater prevalence of 11.8% for men compared to 8.5% for women [1]. The overall estimated increase with age was 1.94 per 10-year increment. In Germany, the prevalence in persons from 45 to 64 years is 5.7% and in persons older than 64 years 11.7%. As in younger persons, beyond 64 years, men are more frequently affected than women (in Germany 12.5% versus 11.0%) [2]. According to the World Health Organization, COPD in 2030 will be the third most frequent cause of death worldwide. Smoking is the most frequent risk factor for COPD, followed by pollution and occupational hazards [3].

Inhaling long-acting anticholinergic and β-adrenergic drugs (in severe cases combined with inhaled glucocorticoids) represent the cornerstones of treatment of pulmonary obstruction caused by COPD. Many elderly patients make severe mistakes which impede adequate drug delivery to the bronchioles. Most drugs for the treatment of COPD are inhaled either via metered dose inhalers (MDI) with the drug as a gas, soft mist inhalers (SMI, trade mark Respimat) or dry powder inhalers (DPI). Elderly patients suffering from COPD, particularly those with impaired vision, cognitive decline or motor/sensory disturbances of their fingers, encounter difficulties in handling inhaler devices used as the cornerstones of treatment of pulmonary obstruction.

Adherence to the prescribed treatment plan is low in COPD patients. In studies the reason was either forgetfulness or deliberate omission of one or more doses. Patient compliance decreased with the number of drugs prescribed [4–7]. Intensive counselling and an empathic interaction between physician and patient were able to improve compliance [8]. Moreover, adherence was highest in patients inhaling only once every 24 h. Adherence to the prescribed treatment protocol reduced the frequency of exacerbations and of hospital admissions thereby increasing the quality of life of COPD patients [9, 10]. The second problem of COPD therapy is inappropriate handling of the inhaler devices. In a study on 188 patients with COPD and 112 patients with bronchial asthma using similar inhaler devices, only 17.7% of the participants inhaled correctly, whereas 94.3% of the MDI and 82.3% of DPI users made at least one mistake [11]. In a more recent study on 103 COPD patients, 74.8% of the participants made at least one essential mistake during the inhalation of the drug [12]. According to previous studies, no inhalator device was substantially superior to others. Patients were unable to judge whether they used their inhaler correctly [13]. The frequency of handling mistakes increased with the age [14].

The present study was driven by the observation on our clinical rounds that the frequency of treatment protocol violations and of inhaler device handling mistakes was extremely high among the patients treated in our geriatric department. This inspired us to set up an 8 day systematic training aiming at improving the adherence to the prescribed treatment protocols and reducing the number and severity of inhaler handling mistakes.

## Methods

### Patients

From October 1, 2016 to September 30, 2017, we conducted a single-center, non-randomized, open prospective intervention study in the Dept. of Geriatrics, Protestant Hospital Göttingen-Weende. We included patients ≥65 years with previously diagnosed stable COPD, who used at least one inhaler device regularly and independently and had been using this device prior to hospital admission, were able to communicate in German without problems and gave their written informed consent to participate. We did not include patients with an acute exacerbation of their COPD or with another diagnosis potentially causing dyspnea (e.g., rib fractures, acute cardiac failure, acute pulmonary embolism, severe pain during degenerative diseases of the spinal cord or vertebral fractures, previous diagnosis of bronchial asthma). COPD exacerbations are heterogeneous events associated with increased airways and systemic inflammation. We excluded them, since during exacerbations lung physiology parameters and CAT scores change rapidly precluding an assessment of the effect of the intervention. Furthermore, we did not include patients considering themselves as non-compliant.

### Study protocol

The study consisted of a) standardized assessments of the handling of the inhaler(s) to be performed by M.C.L., b) a questionnaire measuring the symptom load to be filled out by the patient, and c) a body plethysmography before and after the intervention, i.e., on day 1 and day 8. The standardized check lists for each individual inhaler device were developed by Dr. Verena Knipel and Prof. Dr. Wolfram Windisch, Lungenklinik Merheim, Köln, University of Witten-Herdecke, in 2013. These check lists are freely available on the homepage of Deutsche Atemwegsliga e.V. (German Airway League; https://www.atemwegsliga.de/atemwegsliga.html). To reliably score the inhaler devices of different companies, for each inhaler 10 steps of the inhaling procedure were defined and scored either as correct or wrong. For the quantification of the patient’s symptom load the COPD Assessment Test (CAT Score) (https://www.atemwegsliga.de/copd-assessment-test.html) was used consisting of 8 individual questions on COPD symptoms, which the patient had to quantify on a scale from 0 to 5 [15]. Body plethysmography was performed by Jäger MasterScreen™ Body (CareFusion, Leibnizstrasse 7, 97,204 Hoechberg, Germany). For the pre/ post intervention comparison, the volume expired by forced expiration in 1 sec (FEV1), the Tiffeneau Index [FEV1/ forced vital capacity (FVC)] and the FVC were used [16]. On day 1, the patient also underwent a short structured interview, and his demographic data and his cognitive status (Mini Mental Status Test; MMST) were recorded. After the completion of the entry assessment, from day 2 to 7 an intensive video-assisted training was started: every day the patient watched the video clip demonstrating the correct handling of his inhaler device. The video clips were developed by Marcus Gloger, iKomm GmbH, Bonn, under the scientific supervision of Prof. Wolfram Windisch and Dr. Verena Knipel, from 2011 to 2013 [17]. They can be downloaded from the homepage of Deutsche Atemwegsliga (“Richtig inhalieren - Deutsche Atemwegsliga e.V.”; https://www.atemwegsliga.de/richtig-inhalieren.html). For each type of inhaler, an adequate movie was developed lasting from 1:47 min to 2:47 min [17]. Thereafter, the patient was daily encouraged to use his own inhaler device under the supervision and with verbal and non-verbal aids by his trainer. A typical daily training session with the trainer lasted 6–7 min.

### Statistics

Comparisons were performed by Graph Pad Prism 6 (GraphPad Software Inc., San Diego, CA, USA) by non-parametric methods (two-tailed Wilcoxon signed rank test for pre/post intervention comparisons, Mann-Whitney U test and Kruskal-Wallis test for unpaired observations). When appropriate, correction for repeated testing was performed by the Bonferroni method. For the analysis of correlations, Spearman’s rank correlation coefficient was used. *P* values < 0.05 were considered statistically significant.

### Ethics

The Ethic Committee of the University Medicine Göttingen, Germany, consented in the conduction of this study. Before giving their informed consent, participants were informed in written form and orally about the purpose and the course of the study. Data were immediately pseudonymized, and data analysis was conducted after anonymization.

## Results

Forty-one patients (22 female, 19 male) were included in this study (Table 1). Three patients (all female) dropped out. One patient was discharged before he had completed the training, one patient was unable to complete the assessment because of gastroenteritis, and one patient died prior to the end of the training program. These patients were not further evaluated, i.e., 19 women and 19 men completed the study. Median age was 79.0 years (range: 66–93; median women 79.0 years, men 79.0 years). The mean age ± standard deviation (SD) of the participants of this study was 79.1 ± 6.0 years (women 79.8 ± 7.2 years, men 78.3 ± 4.6 years) compared to a mean age of approx. 82 years of all patients treated in our department. According to the classification of the Global Initiative of Chronic Obstructive Lung Disease (GOLD), 9 patients were classified as GOLD I, 17 as GOLD II, 12 as GOLD III, and none as GOLD IV. As inhaler, 3 patients used a MDI, 26 a DPI (11x Breezhaler™, 6x Handihaler™, 6x Novolizer™, 2x Aerolizer™, 1x Turbohaler™), and 9 a Respimat™, a SMI. In 18 patients on day 1 FEV1/ FVC was < 0.7. In 22 patients, the Mini Mental Status Test was performed as part of the geriatric assessment. An abnormal score (≤ 26) suggesting cognitive impairment was found in 16 patients. The principal diagnoses upon admission were traumatic and degenerative bone and joint diseases (*n* = 12), pneumonia and bronchitis (*n* = 10), other infections (*n* = 3), heart failure of different origins (*n* = 7), gastrointestinal diseases (*n* = 3), acute renal failure (*n* = 2), and neurological diseases (*n* = 1). Nineteen patients were in the acute and 19 in the rehabilitation phase. No violations of standards of testing during the performance of body plethysmography were noted. The mean duration of the stay of our in-patients was approx. 16 days.

**Table 1:** Summary of clinical data of the patients studied

		Number (n)
Patients completing the study	Male	19
	Female	19
Age (years)	Total cohort [median (minimum - maximum)	79.0 (66–93)
FEV1/ FVC < 70% on day 1 (*n* = 18)	Male	10
	Female	8
Mini Mental Status Test performed (*n* = 22)	Cognitive decline	16
	Normal cognition	6
Classification GOLD	I	9
	II	17
	III	12
	IV	0
Classification COPD	A	3
	B	23
	C	0
	D	12
Type of inhaler device	MDI	3
	DPI	26
	SMI (Respimat™)	9
Drop outs	–	3

The median number of mistakes upon entry in this study was 3.0 (minimum - maximum: 0–7 mistakes). The medians for the individual inhalers were: MDI 3.0; DPI 2.5 (Breezhaler™ 4.0, Handihaler™ 4.0, Novolizer™ 2.0); SMI 4.0. For each type of device the most common mistakes were a) insufficient expiration prior to inspiration and b) lack of holding the breath for 5–10s after inhalation.

**Fig. 1:**
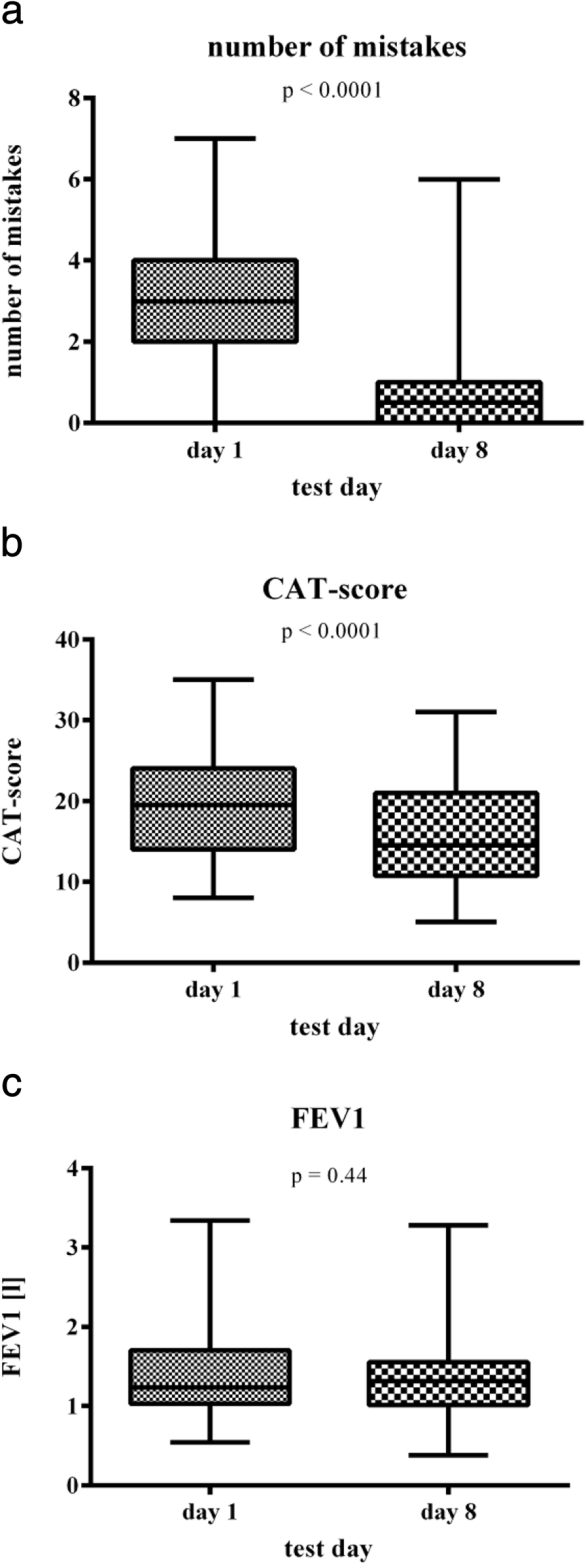
Number of mistakes (**a**), of the intensity of perceived COPD symptoms (COPD Assessment Test, CAT Score) (**b**) and of the pulmonary obstruction (forced expiratory volume in 1 s, FEV1) (**c**) prior and after an 8-days intensive multimodal training. The number of mistakes (**a**) and the COPD symptom load (**b**) were strongly reduced by the training program. Conversely, FEV1 as an indicator of pulmonary obstruction (**c**) virtually remained unchanged. Data are presented as medians (large horizontal bars), 25th/75th percentiles (lower and upper edges of the squares), and minima and maxima (small horizontal bars)

On day 1, only 2 patients (5.3%) inhaled without mistakes. On day 8, the median number of mistakes was 0.5 (minimum – maximum: 0–6 mistakes) (difference versus day 1: *p* < 0.0001) (Fig. 1a). After completion of the training program, 19 patients (50%) inhaled without mistakes. Thirteen patients (34.2%) made one mistake, i.e., 84.2% made ≤1 mistake. The median reduction of mistakes was comparable for the individual inhalers used: MDI 3.0; DPI 2.0 (Breezhaler™ 2.0, Handihaler™ 2.0, Novolizer™ 2.0); SMI 3.0.

Upon entry in this study, the median CAT Score was 19.5 (minimum – maximum: 8–35). On day 8, after completion of the training program, the median CAT Score was 14.5 (minimum – maximum: 5–31). In median, the reduction of the CAT Score from day 1 to day 8 was 4.0 (*p* < 0.0001) (Fig. 1b). The median reduction of the CAT Score was similar with the individual inhalers with the exception of the MDIs, which were used in only 3 patients: MDI ±0; DPI 4.0 (Breezhaler™ 4.0, Handihaler™ 4.5, Novolizer™ 4.5); SMI 4.0. The FEV1 as a means of pulmonary obstruction in median slightly increased, the difference did not reach statistical significance (median FEV1 day 1: 1235 ml; median FEV1 day 8: 1315 ml) (*p* = 0.44) (Fig. 1c). The median FVC also slightly increased [median FVC day 1: 1720 ml (25th/75th percentile 1370/2520 ml); median FVC day 8: 1850 ml (25th/75th percentile 1410/2590 ml)], the difference again was not statistically significant (*p* = 1.0).

The separate analysis of the patients with a FEV1/FVC < 70% (i.e., patients with a pulmonary obstruction typical for COPD; *n* = 18), in median, revealed a reduction of the number of mistakes from 2.0 (minimum – maximum: 0–6) to 0.5 (minimum – maximum: 0–5) (*p* < 0.0002). The median reduction of the CAT Score in this subgroup was 3.5 (*p* = 0.0088). The median FEV1 showed a small increase from day 1 (1185 ml) to day 8 (1280 ml), which also failed to reach statistical significance (*p* = 0.46). The median FVC also increased [median FVC day 1: 1830 ml (25th/75th percentile 1510/2850 ml); median FVC day 8: 2110 ml (25th/75th percentile 1710/2780 ml)], the difference, however, was not statistically significant (*p* = 0.28). Subgroup analyses according to sex and type of inhaler device used showed comparable reductions of the numbers of mistakes and of the CAT Scores in each subgroup, and the FEV1 did not change significantly in all subgroups.

According to the Global Initiative for Chronic Obstructive Lung Disease (GOLD) criteria, 9 patients were classified grade I, 17 patients grade II, 12 patients grade III, and no patient grade IV. The number of mistakes was reduced in GOLD grades I – III (*p* ≤ 0.03). The CAT Score improved significantly in patients classified GOLD grade II (*p* = 0.004), whereas in the other groups there was a tendency towards the relieve of symptoms (GOLD grade I: *p* = 0.12; grade III: *p* = 0.14). The FEV1 did not change substantially in all GOLD grades studied.

The number of handling mistakes on day 1 was inversely correlated with the MMST Score (*n* = 22; r_S_ = − 0.52; *p* = 0.013), i.e. patients suffering from cognitive decline upon entry into this study made more mistakes than patients with normal cognition. Conversely, the reduction of mistakes did not depend on the MMST Score (r_S_ = 0.017; *p* = 0.94), i.e., patients with normal and abnormal cognition equally benefitted from the training program. Only one patient with a MMST Score of 14 was not able to reduce the number of mistakes during inhaling suggesting that patients with severe cognitive deficits may not have a benefit from this exercise program. The greatest reduction of inhaling mistakes (reduction by 4 mistakes) was seen in two patients with mild cognitive decline (MMST Score 25 and 26) suggesting that this group of patients definitely showed a benefit from training. Age did not significantly correlate with the number of mistakes on day 1 and with the reduction of the number of mistakes from day 1 to day 8 (r_S_ = 0.19 and 0.17; *p* = 0.26 and 0.32).

## Discussion

In geriatric patients the effectiveness of inhaler therapy for COPD is impaired by many mistakes. In this study, the number of mistakes was strongly reduced by an intensive video-assisted training of 8 days (reduction of the median number of mistakes from 3.0 to 0.5) irrespective of the inhaler device used. This corresponded with a strong reduction of the severity of perceived symptoms as measured by the difference of the CAT Score at entry and after completion of this study. A reduction of the CAT Score by 2 points has been considered the minimal clinically important difference (MCID) in previous studies [18], i.e., the reduction of the median CAT Score by 4 points in the present study is of importance for the well-being of our patients. The benefit was approximately equal over the full age range tested (66–93 years) and in cognitive normal and abnormal patients. FEV1 remained virtually unchanged reflecting the nature of COPD as a non-reversible degenerative disease of the lung.

Although the patients had used the inhaler device already at home, in the first assessment, 36 of 38 patients made at least one mistake in the use of this device (94.7%). Other studies came to similar results: in a study on 52 patients with COPD or bronchial asthma, only 6% used their inhaler correctly [19]. Similarly, only 13% of 296 adults (age 50–92 years) with COPD who had been prescribed a MDI used this device correctly [1]. In a recent study, where 103 patients using MDIs or DPIs were included, 74.8% made at least one essential mistake [12]. In previous studies, upon study entry older and cognitively impaired patients made more mistakes than younger patients [20–22]. Here, the number of handling mistakes on day 1 was increased in patients with cognitive decline, whereas it did not correlate with age.

The 8 day-intervention led to a strong improvement of the handling of the inhaler devices (in median 0.5 versus 3 mistakes), irrespective whether the patients used MDIs or DPIs or a Respimat™. Several studies investigated the effectiveness of different training programs. Generally, a multimedia intervention accompanied by verbal comments was more effective than an exclusive written or verbal explanation [13, 19, 23]. A meta-analysis, however, concluded that especially for geriatric patients it remains unclear, whether verbal or written instruction or video movies are more effective [14]. Unlike other interventions, our intense training program was equally effective over the whole age range studied and in cognitive normal and abnormal patients. The strong reduction of mistakes observed in the present study irrespective of the age and cognitive status of the patient suggests that the daily combination of video material, physical demonstration and verbal instructions is most effective. The independence of the number of mistakes on day 1 and of the training effect from the age range of 66–93 years suggests that age is no contraindication for the use of inhaler devices: even the oldest-old (≥ 85 years) [24] did not make more mistakes on day 1 than younger patients and benefitted from training in its present form. The fact that the greatest reduction of inhaling mistakes was seen in two patients with mild cognitive decline suggested that our multimodal approach containing redundant information repeatedly presented via several channels may be particularly effective in patients with mild cognitive decline.

This study has several limitations, in particular its design as a single-center, non-randomized, unblinded intervention study without a control group. We did not assess the effect of training on the resolution of symptoms during an acute exacerbation, and we excluded patients considering themselves as non-compliant. Moreover, the number of participants was relatively low: although the reduction of the number of mistakes was comparable with all inhaler devices used, the low number of patients using individual inhalers precluded the analysis of subgroups of patients using individual inhalers. We did not conduct a follow-up of the patients. For this reason, it is unclear, for which interval this training program was effective. Studies in younger patients had shown that the effect of training lasted up to 3 months [22, 25]. It remains to be studied, whether this is also true for old patients without and with cognitive impairment. A recently started Portuguese randomized study using teach-to-goal placebo-device training versus usual care and a 1-year follow-up in patients > 65 years of age with asthma or COPD with several interim analyses will probably help to answer the question of the duration of the training effect in old patients soon [26].

Since the physicians in charge of the patients had to judge the potential participant of this study as capable of giving informed consent before study entry, in this study many patients with mild cognitive decline and no patient with a MMST Score below 14 were included. Therefore, we cannot answer the important question whether training in the present form is effective in patients with severe dementia. Here, a tailored teach-to-goal placebo device training, which allows many repetitions, may be more effective. This question probably will also not be answered by the Portuguese study, since it also requires written informed consent from each participant at entry into the study and thereby excludes patients who are unable to give their informed consent [26].

The main strengths of this study were the inclusion of patients ≥65 years only, the high age of the participants, the exclusion of patients with bronchial asthma and the intensity of the training program leading to a strong reduction of the numbers of mistakes independent of age and cognitive status. Furthermore, the intense training led to a strong reduction of the patients` perceived symptom load as measured by CAT Score. The FEV1 and the FVC increased slightly, the difference failed to reach statistical significance. Patients with a severe decline of their lung function may in fact have some potential benefit from small increases of the FEV1 and FVC. The sample size of the present study resulted in a low statistical power to detect such small differences. The failure to strongly improve the FEV1 and FVC by correct application of the inhalers for 1 week was not unexpected. COPD is a chronic pulmonary degenerative disease usually associated with mild inflammation, and the full effect on pulmonary obstruction usually is seen after several weeks of therapy [27, 28].

## Conclusion

In COPD, intensive multimodal training including daily short video movies, demonstration and counselling for 8 days improved the handling of inhaler devices and reduced clinical symptoms. No significant reduction of airway obstruction was noted in a group of true geriatric patients. Patients with cognitive abnormalities also had a benefit from the intervention. It remains to be studied, how long the training effect will last and whether also patients with severe cognitive abnormalities will benefit from such program.

## Data Availability

The anonymized data forming the basis of this manuscript will be made available to others upon reasonable request by the corresponding author.

## Abbreviations

CA: California
CAT: COPD Assessment Test
COPD: Chronic-obstructive pulmonary disease
DPI: Dry powder inhaler
FEV1: Forced expiratory volume in 1 s
FVC: Forced vital capacity
GOLD: Global Initiative for Chronic Obstructive Lung Disease
MCID: Minimal clinically important difference
MDI: Metered dose inhaler
MMST: Mini Mental Status Test
USA: United States of America
SMI: Soft mist inhaler
